# All-trans retinoic acid enhances anti-proliferative effect of dual PI3K and mTOR inhibitor NVP-BEZ235 in triple negative breast cancer

**DOI:** 10.1007/s00210-025-03981-8

**Published:** 2025-03-05

**Authors:** Suranur Ayvaz, Zeynep Busra Bolat

**Affiliations:** 1 Molecular Biology and Genetics Department, Hamidiye Institute of Health Sciences, University of Health Sciences, Istanbul, 34668 Türkiye; 2 Experimental Medicine Research and Application Center, Validebag Research Park, University of Health Sciences, Uskudar, Istanbul, 34662 Türkiye; 3 Department of Molecular Biology and Genetics, Faculty of Engineering and Natural Sciences, Istanbul Sabahattin Zaim University, Istanbul, 34303 Türkiye

**Keywords:** NVP-BEZ235, All-trans-retinoic acid, Triple negative breast cancer, Anti-proliferative effect

## Abstract

Breast cancer remains the most diagnosed cancer in females and one of its most challenging subtypes is Triple Negative Breast Cancer (TNBC). Treatment of TNBC presents challenges due to limited targeted therapies, inefficacy of chemotherapy, and severe side effects. Therefore, combination therapies are preferred to reduce toxicity and drug resistance. All-trans-retinoic acid (ATRA), a key player in cell growth, differentiation, and organogenesis, also exerts significant anti-cancer effects. NVP-BEZ235 is a dual PI3K and mTOR kinase inhibitor. In this study we investigated the anti-proliferative potential of NVP-BEZ235 and ATRA on TNBC cell line MDA-MB-231. The effective combination dosage was found to be 1 µM for NVP-BEZ235 and 5 µM for ATRA on MDA-MB-231 cells at 48 h. Combination treatment of NVP-BEZ235 and ATRA significantly reduced migration and colony formation compared to the control group. Co-treatment of NVP-BEZ235 and ATRA showed increase at G0/G1 phase in MDA-MB-231 cells. Treatment of NVP-BEZ235 and ATRA in MDA-MB-231 cells showed a significant increase in Caspase-3 genes, while a significant decrease in mTOR and BCL-2 genes were detected when compared to the untreated group. These results indicate that this combination therapy is a promising anti-cancer agent and has potential use in the treatment of TNBC.

**Supplementary Information:**

The online version contains supplementary material available at 10.1007/s00210-025-03981-8.

## Introduction

Breast cancer is the most frequent cancer in females worldwide and is one of the leading causes of cancer-related deaths (Bray et al. 2024). Breast cancer is a heterogeneous disease and is generally classified into three subtypes according to receptor expression: estrogen and progesterone receptor positive (ER + /PR +), human epidermal growth factor receptor positive (HER2 +), and triple negative (ER-/PR- /HER2 −). Triple Negative Breast Cancer (TNBC) accounts for 20% of all breast tumors and is the most aggressive subtype of breast cancer (Bao and Prasad 2019). Treatment of TNBC is very difficult due to the small number of targeted therapies, the poor prognosis of chemotherapy, and high side effects. Combination therapies strategy is generally preferred in the treatment of TNBC to overcome toxicity and drug resistance (Lin et al. 2017; Paroni et al. 2020).

Retinoids, synthetic or natural analogues of vitamin A, play role in cancer development, cell growth and cell differentiation (Wang et al. 2013). ATRA, an active metabolites of vitamin A, shows anti-cancer agent properties besides its role in cell growth, differentiation and organogenesis processes (Mezquita et al. 2018; Giuli et al. 2020). Studies have reported that ATRA induces apoptosis and inhibits cell cycle progression in breast cancer MDA-MB-231, MCF-7, SK-BR-3 and HCC1806 cell lines (Wang et al. 2013; Lin et al. 2017). Retinoids derivatives have also been used in clinical studies to investigate the therapeutic effect in human breast cancer (Howe 2007). It has also been reported to inhibit mammary carcinogenesis in animal models (Lin et al. 2017). Retinoids exert their biological effects through a variety of mechanisms mediated by interactions with the two identified families of retinoid nuclear receptors, retinoic acid receptors (RARs) and retinoid X receptors (RXRs). Activated receptor complexes regulate the expression of target genes (Howe 2007). ATRA-mediated gene expression can affect cell differentiation, proliferation, apoptosis and metabolism (Howe 2007; Siddikuzzaman et al. 2011; Chlapek et al. 2018; Moosavi and Djavaheri-Mergny 2019). It has been reported that ATRA affects the growth and proliferation of cancer cells by affecting MAPK, mammalian target of Rapamycin (mTOR) and indirectly phosphatidylinositol-3-kinase (PI3K)/Akt pathways (Giuli et al. 2020).

PI3K, a lipid kinase, plays a central role in cell growth, proliferation, survival and regulation of metabolism (Schnell et al. 2008). PI3K binds to the activated tyrosine kinase receptor at its SH2 unit, resulting in the conversion of phosphatidylinositol-4,5-biphosphate (PIP2) to phosphatidylinositol-3,4,5-trisphosphate (PIP3). AKT binds to PIP3 and localizes to the plasma membrane. PIP3 also activates Phosphoinositide-dependent kinase-1 (PDK1), resulting in AKT activation by the phosphorylation of PDK1 and mTOR. Activated AKT phosphorylates multiple downstream targets involved in cell survival, protein synthesis and metabolism (Porta et al. 2014; Yang et al. 2014). Disruptions in the PI3K/Akt pathway are among the most common problems in different types of human cancer. This has made PI3K an attractive therapeutic target for inhibitors to be used in cancer therapy (Serra et al. 2008; Liu et al. 2009).

NVP-BEZ235, imidazoquinoline derivative, that is a dual PI3K and mTOR kinase inhibitor (Maira et al. 2008; Torki et al. 2017) binds to the ATP binding site of kinases and competes with ATP, reducing enzyme activity reversibly (Serra et al. 2008). NVP-BEZ235 showed anti-tumor activity by inhibiting the growth of colorectal, globlastoma and breast cancers (Maira et al. 2008). Treatment of NVP-BEZ235 alone slowed tumor growth rate and reduced metastasis in osteosarcoma, Ewing's sarcoma and alveolar rhabdomyosarcom (Manara et al. 2010). Combination treatment of NVP-BEZ235 with conventional cytotoxic agents maximize its therapeutic potential and show promise in cancer studies (Maira et al. 2008; Manara et al. 2010). Also, NVP-BEZ235 shows synergistic effect with other polyphenols in neuroblastoma (Çetin et al. 2023), renal carcinoma (Seo et al. 2014) and breast cancer (Torki et al. 2017).

The purpose of our study was to increase the efficacy of NVP-BEZ235 on TNBC MDA-MB-231 cell line by using this chemotherapeutic agent as combination therapy with ATRA. Cell viability of MDA-MB-231 cells were investigated under the treatment of NVP-BEZ235, ATRA or their combination. The anti-cancer efficacy of NVP-BEZ235 and ATRA was assessed through cell cycle, colony formation assay, invasion assay and apoptotic related gene expression analysis.

## Material & method

### Cell culture and reagents

Human TNBC cell line MDA-MB-231 (HTB-26, ATCC) was maintained in RPMI-1640 media supplemented with 10% fetal bovine serum (FBS, Invitrogen, Gibco, UK) and 100 units/mL of penicillin, 100 μg/mL of streptomycin and amphotericin (1% PSA, Invitrogen, Gibco, UK). The human normal epithelial mammary cell line MCF-10A (CRL-10317, ATCC) was cultured in MEGM kit medium (Lonza, CC3150) supplemented with 2% FBS and 1% PSA. Both cells were incubated in a humidified atmosphere with 5% CO_2_ at 37 °C. ATRA was purchased from Stem cell technologies (Canada). NVP-BEZ235 was supplied by Selleck Chemicals (USA) and dissolved in DMSO. The DMSO concentration of reagents were less than 0.1% in all experiments.

### Cell viability assay

Cell viability was determined using the 3-(4,5-dimethylthiazol-2-yl)−5-(3-carboxy-methoxyphenyl)−2-(4-sulfo-phenyl)−2H-tetrazolium (MTS) colorimetric assay (CellTiter96 AqueousOne Solution; Promega, UK). In 96-well plates, MDA-MB-231 and MCF-10A cells were seeded with a density of 5 × 10^3^ cells/well. After 24 h (h), cells were treated with 0.1, 0.2, 0.5, 1, 2, 5, 10 µM NVP-BEZ235, 0.5, 1, 2.5, 5, 10, 20, 40 µM ATRA or their combination for 24 h, 48 h and 72 h. Following post treatment, cells were subjected to MTS assay according to manufacturer’s protocol and absorbance was measured at 490 nm using a microplate reader (Bio-tek ELx800, USA). Cell viability (%) was determined by setting non-treated control cells to 100%.

### Colony formation assay

Colony formation assay was performed to measure the reproductive viability of MDA-MB-231 cells as described previously (Yao et al. 2019). Briefly, cells were seeded in 6-well plates at a density of 1000 cells/well. After 24 h, when the cells were attached, the medium was replaced with a medium containing 5 µM ATRA, 1 µM NVP-BEZ235 and their combinations, followed by an incubation of 12 days. Then, colonies were fixed with 100% methanol for 20 min at + 4 °C and stained with 0.1% crystal violet for 5 min at room temperature. The stained colonies in each well were visualized with a light microscope (Primovert, Zeiss) and then analyzed by counting the colony numbers.

### Invasion assay

The transwell insert (Corning, ABD) was coated with 100 µL of 1 mg/mL Matrigel (Corning, USA). Cells were resuspended in serum-free RPMI 1640 medium (5 × 10^4^/100 µL), added to the insert, while the wells contained 5 µM ATRA, 1 µM NVP-BEZ235 or their combinations supplemented with 10% FBS. After 48 h, cells that migrated to the wells were fixed with 100% methanol, stained with 0.1% cristal violet for 5 min at room temperature and washed with PBS. Cells were counted under the microscope (Primovert, Zeiss) with camera.

### Cell cycle assay

In 6-well culture plates, MDA-MB-231 cells were seeded at a density of 1.5 × 10^5^ cells/ well. The following day, the cells were subjected to 5 µM ATRA, 1 µM NVP-BEZ235, or a combination of both. After 48 h incubation, both adherent and non-adherent cells were collected, thoroughly washed with PBS, and subsequently fixed with 70% ethanol at −20 °C for at least 2 h. Following fixation, samples were centrifuged, washed with PBS and stained with a 200 μL solution of Tali® Cell Cycle (Thermo Fisher, USA) solution containing a mixture of propidium iodide (PI), RNase A, and Triton X-100, for a duration of 30 min. The samples prepared were assessed by obtaining data from 20,000 events using flow cytometry (CytoFLEX, Beckman Coulter, USA).

### Quantitative PCR (qPCR)

MDA-MB-231 cells were seeded in 6 well plates with a density of 1.5 × 10^5^ cells/well. Cells treated with 5 µM ATRA, 1 µM NVP-BEZ235, or a combination of both for 48 h. Pellets were collected for isolation of total RNA using TRIZOL (VWR Life Science, TriFast) according to the manufacturer's instructions. cDNA was synthesized from the isolated total RNA using One-Step cDNA kit (ABMgood) according to the manufacturer's protocol. SYBR Green Master Mix (Nepenthe) was used for the qPCR to quantify mRNA levels of the genes. Primers were designed using Primer-BLAST software from the National Center for Biotechnology information (USA) and synthesized by Sentebiolab (Turkiye). Primers sequences were given in Table S1. 18SrRNA (QIAGEN) was used as a housekeeping gene to ensure equal loading and the data were analyzed using 18SrRNA for normalization of control. qPCR experiments were conducted using CFX96 RT-PCR system (Bio-Rad, Hercules, CA, United States). The results were normalized to 18SrRNA mRNA levels and the relative fold change values were analyzed using the delta delta Ct (2^−ΔΔCt^) method.

### Statistical analysis

All data (*n* = 3) were presented as mean ± standard deviation (SD) and GraphPad Prism version 8.2.1 (San Diego, USA) were used for all statistical analysis. Statistical significance was determined by student t-test, one-way or two-way ANOVA followed by a post-hoc Tukey test when necessary. *P* values were set as *p* < 0.05 (*), *p* < 0.01 (**), *p* < 0.001 (***) and *p* < 0.0001 (****).

## Results

### ATRA and NVP-BEZ235 inhibits the cell proliferation of MDA-MB-231 cells

**Fig. 1:**
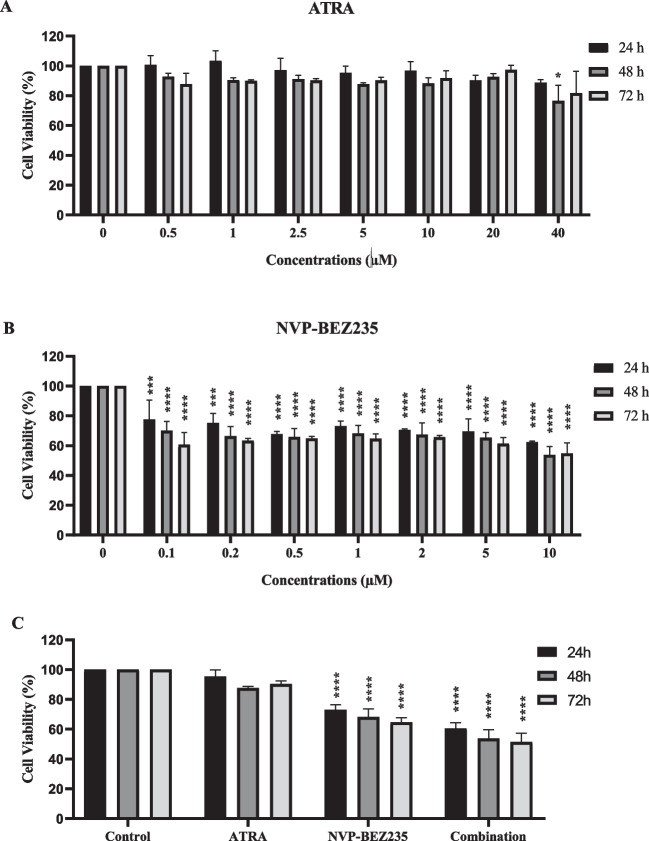
Effects of ATRA, NVP-BEZ235 and their combination on the MDA-MB-231 cell line. Percentage of viable MDA-MB-231 cells after treatment with **A** ATRA, **B** NVP-BEZ235 and **C** their combination for 24, 48 and 72 h. The cell viability was determined by MTS assay. Data are presented as mean ± standard deviation of three independent studies (*n* = 3) (*p < 0.05, **p < 0.01, ***p < 0.001, ****p < 0.0001)

We evaluated the viability of MDA-MB-231 cells treated with ATRA, NVP-BEZ235, and co-treatment (ATRA and NVP-BEZ235) for 24, 48 and 72 h using the MTS assay. Our results demonstrated that MDA-MB-231 cells treated with 0.5, 1, 2.5, 5, 10, 20 and 40 µM ATRA showed no significant decrease (*p* > 0.05) in cell viability except for highest dose (40 µM ATRA) at 48 h, where a significant decrease in cell viability to 76.68 ± 10.34% was detected. At 72 h, it was observed that the MDA-MB-231 cells recovered for 40 µM ATRA (Fig. 1A). On the other hand, treatment with NVP-BEZ235 decreased cell viability significantly in a dose- and time-dependent manner (Fig. 1B). At 48 h, MDA-MB-231 cells treated with 0.1, 0.2, 0.5, 1, 2, 5, and 10 µM NVP-BEZ235 showed significant decrease of cell viability to 70.13 ± 6.08%, 66.41 ± 6.32%, 65.98 ± 5.50%, 68.20 ± 5.40%, 67.32 ± 8.07%, 65.31 ± 3.42%, and 53.89 ± 5.47%, respectively (Fig. 1B).

The cell viability of 5 µM ATRA combined with different dosages of 0.1, 0.5, 1 and 5 µM NVP-BEZ235 showed a decreased cell viability to 70.48 ± 4.56%, 63.1 ± 5.8%, 53.78 ± 5.87% and 45.21 ± 7.63%, respectively. Thus, 1 µM NVP-BEZ235 and 5 µM ATRA treated MDA-MB-231 cells showed a cell viability of nearly 50% at 48 h (Figure S1). Based on our results, we determined the effective dose of ATRA and NVP-BEZ235 for combination therapy as 5 µM and 1 µM, respectively. At 48 h, MDA-MB-231 cells treated with 1 µM NVP-BEZ235 alone and in combination with ATRA show a decrease in cell viability to 68.20 ± 5.40% and 53.78 ± 5.87%, respectively. Thus, MDA-MB-231 cells treated with combination of 5 µM ATRA and 1 µM NVP-BEZ235 showed a gradual decrease of cell viability to 60.57 ± 3.76%, 53.78 ± 5.87%, and 51.48 ± 5.95% at 24, 48 and 72 h, respectively (Fig. 1C). On the contrary, MCF-10A cells treated with 1 µM NVP-BEZ235 alone and in combination with ATRA showed 70.85 ± 4.18% and 67.94 ± 4.49%, respectively (Figure S2). When compared to MDA-MB-231 cell line, combination therapy showed less toxicity toward healthy epithelial cells, suggesting a possible treatment option for TNBC.

### Combinatorial treatment of NVP-BEZ235 and ATRA decreases colony formation potential of MDA-MB-231 cells

**Fig. 2:**
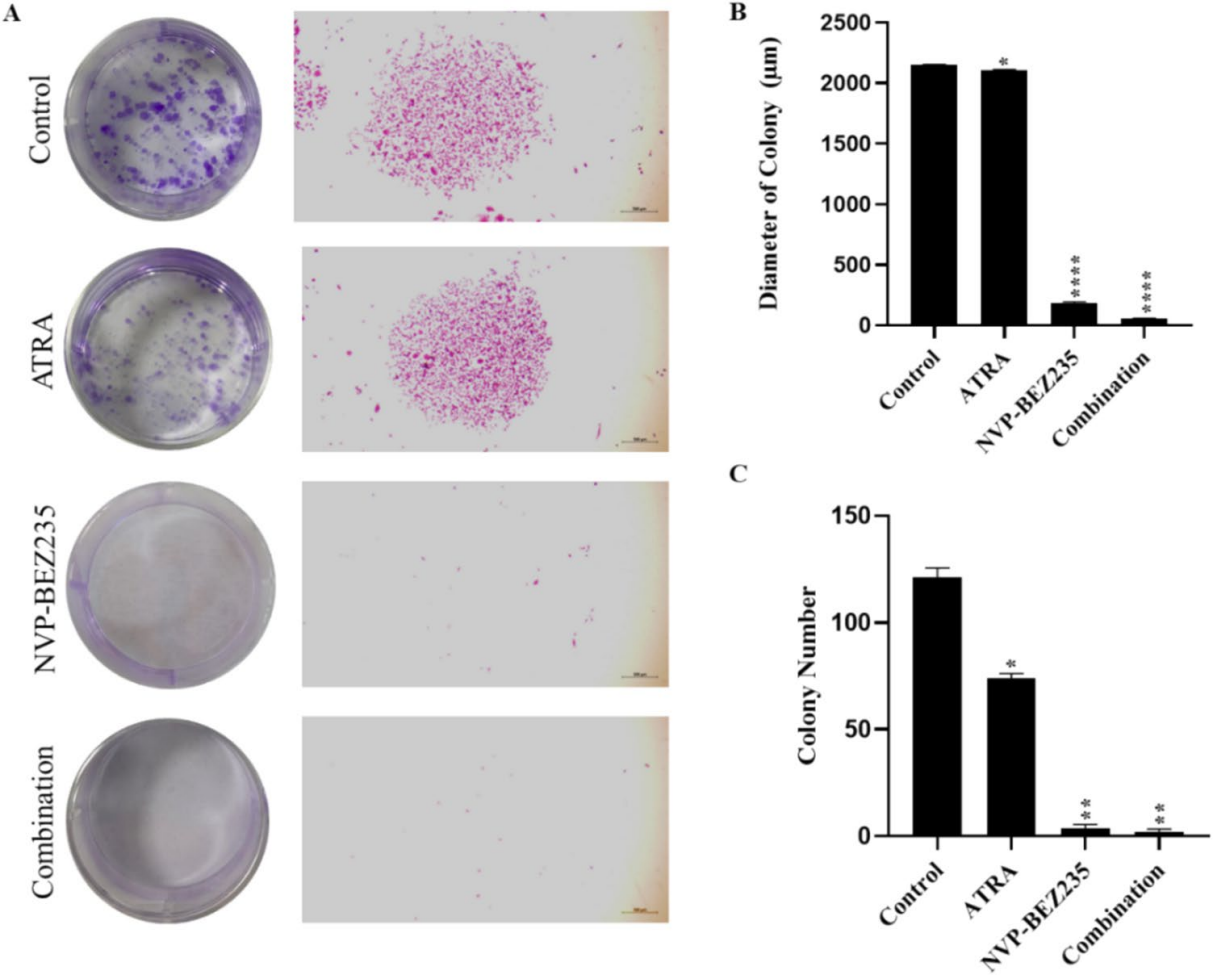
Effect of 5 µM ATRA, 1 µM NVP-BEZ235 and their combinations on colony-forming potential in MDA-MB-231 cells after 12 days. **A** Representative of images of colonies formed in wells (left panel) and colonies of MDA-MB-231 cells taken with a 10X objective (right panel). **B** Colony diameters and **C** Number of colonies for control, ATRA, NVP-BEZ231 and their combination. Data are presented as the mean ± SD of three independent experiments. (*p < 0.05, **p < 0.01, ****p < 0.0001)

A colony formation assay was performed to measure the ability of a single cell to survive and develop into a clonal population over time. The effects of ATRA, NVP-BEZ235 and their combinations on the colony formation abilities of MDA-MB-231 cells were examined. Our results showed that the colony diameters of control group (2149.84 ± 5.38 µm) was significantly reduced in ATRA, NVP-BEZ235, and combinatorial treatment group by 2104.52 ± 8.87 µm, 182.26 ± 11.86 µm and 58.15 ± 1.25 µm, respectively as shown Fig. 2B. Also, colony diameters of ATRA alone (*p* < 0.0001) and NVP-BEZ235 alone (*p* < 0.01) groups showed significant difference to combination group. Similarly, there was a significant decrease in colony number from 121 ± 4.58 (control group) to 74 ± 2, 3.5 ± 2.12 and 2 ± 1.41 in ATRA, NVP-BEZ235 and combination groups, respectively (Fig. 2C). Furthermore, MDA-MB-231 cells when treated with NVP-BEZ235 only showed no significant change (*p* > 0.05) in colony number when compared to combinatorial treatment group.

### NVP-BEZ235 and ATRA inhibits invasion potential of MDA-MB-231 cells

**Fig. 3:**
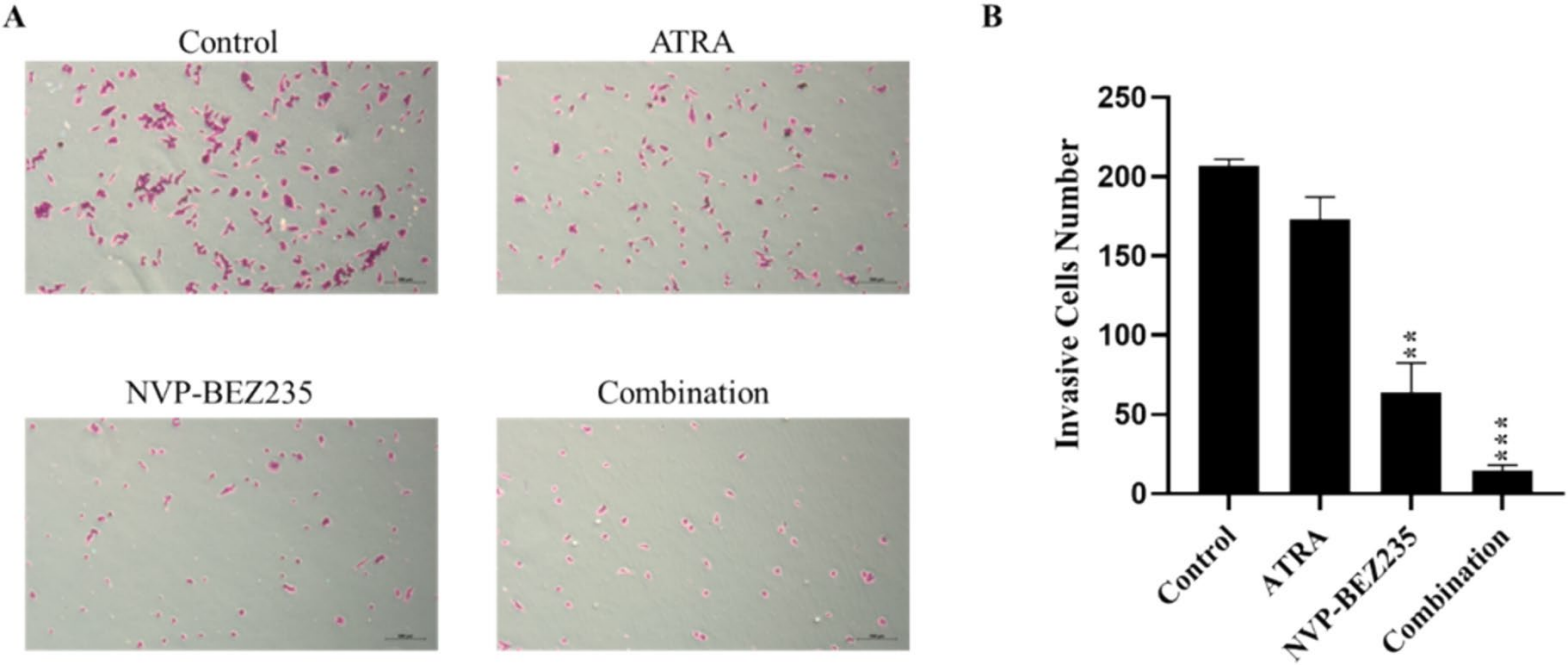
Effect of ATRA, NVP-BEZ235 and their combinations on the invasive potential of cells. **A** Images of invasion assays for cells treated with ATRA, NVP-BEZ235 and their combination, for 48 h. **B** Number of invasive cells. Data are presented as the mean ± SD of three independent experiments. **p < 0.01, ***p < 0.001

An invasion assay was performed to measure the migration of cells through an extracellular matrix. The effects of ATRA, NVP-BEZ235, and co-treatment on the invasion abilities of MDA-MB-231 cells were examined. The control group invasive cells number was 207 ± 4.24. Our results show that ATRA reduced the number of invasive cells to 173 ± 14.14 (*p* > 0.05). However, it is observed that NVP-BEZ235 alone and co-treatment groups significantly inhibits the invasive ability of MDA-MB-231 cells by reducing to to 64 ± 18.38 and 14.5 ± 3.53, respectively (Fig. 3). Furthermore, there was significant change (*p* < 0.05) in the number of invasive MDA-MB-231 cells between NVP-BEZ235 alone and in combination group.

### Co-treatment of NVP-BEZ235 and ATRA increased MDA-MB-231 cell number in G0/G1 phase of cell cycle

**Fig. 4:**
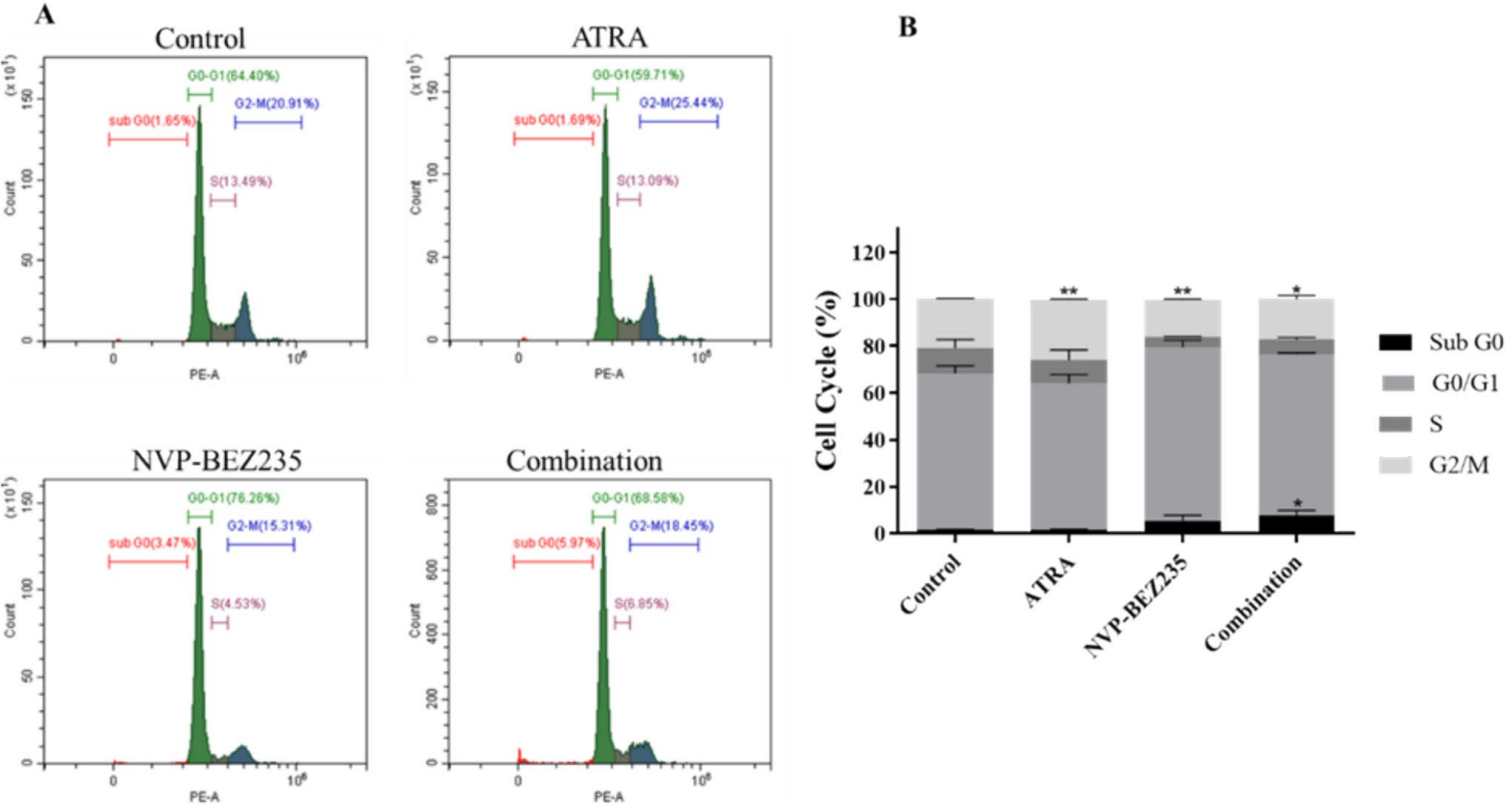
Cell cycle profiles of MDA-MB-231 cells examined by flow cytometry **A** Representative histograms and **B** graphical representation for cell cycle percentage of MDAMB231 cells when treated with control, ATRA, NVP-BEZ235 and their combination showing three different independent repeats (*n* = 3). * *p* < 0.05, ** *p* < 0.01

Flow cytometry was performed to determine the cell cycle progression of MDA-MB-231 cell line when treated with ATRA, NVP-BEZ235 and their combinations (Fig. 4). The frequency of the Sub G0 cell cycle phase (apoptotic cells) were significantly increased after cells were treated with combination of ATRA and NVP-BEZ235 to 7.54 ± 2.22% (*p* < 0.05) compared with 1.5 ± 2.0% in the control group. In ATRA treated MDA-MB-231 cells there was almost no change in Sub G0 phase (1.56 ± 1.8%), while an increase of 5.56 ± 2.5% was detected in NVP-BEZ235 group when compared to control group. MDA-MB-231 cell population in the G0/G1 phase increased from 66.68 ± 3.23% in control cells to 74.11 ± 3.04% and 68.91 ± 0.47% in NVP-BEZ235 and combination groups, respectively (*p* > 0.05). Thus, treatment with NVP-BEZ235, either alone or in combination with ATRA, results in an increased number of MDA-MB-231 cells in the G0/G1 phase of the cell cycle. The frequency of the G2/M cell cycle phase for the control group was 21.03 ± 0.17%, while in the treatment with 5 µM ATRA alone, 1 µM NVP-BEZ235 alone and combination of both were 25.69 ± 0.35%, 15.62 ± 0.43%, and 17.34 ± 1.57, respectively (*p* < 0.05).

### NVP-BEZ235 and ATRA induces apoptotic-related gene expression levels in MDA-MB-231 cells

**Fig. 5:**
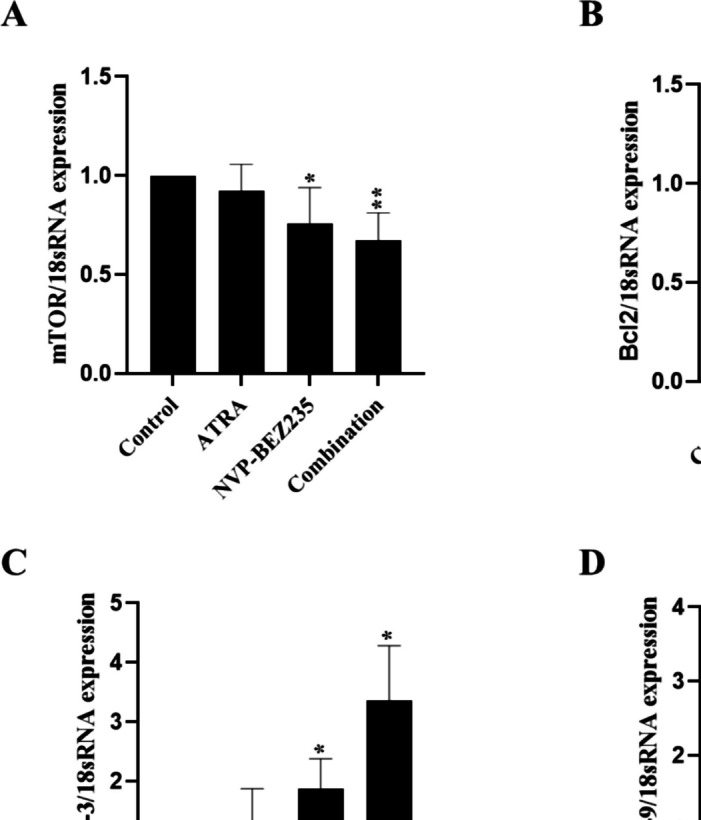
Graphical illustration of the gene expressions levels in MDA-MB-231 cells for **A** mTOR, **B** BCL-2, **C** Caspase-3 and **D** Caspase-9 genes after treatment with 5 μM ATRA, 1 μM NVP-BEZ235, and their combination at 48 h. The presented data shows three independent experiments (*p < 0.05, **p < 0.01, ***p < 0.001)

The relative mRNA expressions for mTOR, BCL-2, Caspase-3 and Caspase-9 of MDA-MB-231 cells treated with ATRA, NVP-BEZ235, and their combinations were analyzed using qPCR after 48 h of treatment. The expression level of mTOR was decreased in the ATRA, NVP-BEZ235, and combination treatment groups compared to the control group, corresponding to a 0.92 ± 0.14, 0.76 ± 0.18 (*p* < 0.05) and 0.67 ± 0.14 (*p* < 0.01) -fold change, respectively (Fig. 5A). As a result of treatment with ATRA, NVP-BEZ235 and their combination, BCL-2 expression level decreased compared to the control group, corresponding to a 0.84 ± 0.16, 0.66 ± 0.30, and 0.63 ± 0.09 (*p* < 0.001) -fold change, respectively (Fig. 5B). The expression level of Caspase-3 was increased in the ATRA, NVP-BEZ235, and combination treatment groups by 1.33 ± 0.55, 1.87 ± 0.51 (*p* < 0.05) and 3.35 ± 0.93 (*p* < 0.05) -fold compared to control group, respectively (Fig. 5C). The Caspase-9 expression level increased in the ATRA, NVP-BEZ235, and combination treatment groups compared to the control group, corresponding to a 1.59 ± 0.19, 2.42 ± 0.72 and 2.74 ± 0.85 -fold increase, respectively (*p* > 0.05) (Fig. 5D). Thus, there was no significant change between ATRA alone and NVP-BEZ235 alone groups and the co-treatment groups gene expression levels except for Caspase-3 gene expression levels, where a significant difference was detected between ATRA alone and combination group (*p* < 0.05).

## Discussion

TNBC therapy focuses on inhibitors that target specific molecular signaling pathways either as single agents or in combination with standard chemotherapy regimens (Costa et al. 2018; Gupta et al. 2020). PI3K/Akt/mTOR signaling pathway, is one of the most common intracellular signaling pathways frequently abnormally activated in various cancer types, including breast cancer (Wu et al. 2022). Development of effective therapeutic agents targeting this abnormality has proven to be a significant challenge due to the complexity of this signaling pathway (Ellis and Ma 2019). Buparlisib (BKM120), a PI3K antagonist, was assessed in a phase 3 trial for TNBC and inhibition of the PI3K pathway alone may be an inadequate therapeutic strategy for TNBC (Garrido-Castro et al. 2020). NVP-BEZ235 is a dual PI3K/mTOR whose mechanism of action is based on promoting cancer cells to apoptosis (Kong and Zhang 2019). It has been reported that this drug, which has low water solubility, may cause specific toxicity to the gastrointestinal system due to accumulation in the intestines, and that it is clinically insufficient when used alone, as it only causes stagnation in the disease (Wise-Draper et al. 2017; Wu et al. 2022). Phase I and phase II clinical trials were discontinued due to adverse side effects such as severe nausea, vomiting, severe fatigue, dose-limiting toxicity, and low tolerability (Fazio et al. 2016). The co-treatment of ATRA and NVP-BEZ235 substantially reduced cell viability of MCF10-A cell line, however this might be overcome in future studies by encapsulating both agents in targeted nanoparticles. Such an approach could decrease drug accumulation in healthy tissues—thereby mitigating adverse gastrointestinal effects and the toxic impact on normal cells—while simultaneously enhancing drug delivery and therapeutic efficacy (Chavda et al. 2023).

Studies using a combination of drugs that inhibit PI3K and its downstream pathway mTOR have shown that first-generation mTOR and PI3K inhibitors have yielded complex results, despite the strength of preclinical data. NVP-BEZ235 has a synergistic effect with Caffeic acid phenyl ester (CAPE) in MDA-MB-231 cells (Torki et al. 2017). ATRA sparked interest in clinical oncology after demonstrating potent therapeutic activity in acute promyelocytic leukemia (Huang et al. 1988; Bollag and Holdener 1992), which lead to the consideration of retinoids as potentially beneficial agents in different solid tumors (Warrell 1994), such as breast cancer. Studies with MCF-7 cells have shown that ATRA reduces cell proliferation (Toma et al. 1997), arrests the G1 phase of the cell cycle (Mangiarotti et al. 1998), and induces apoptosis (Abdolahi et al. 2016). However, studies have demonstrated that ATRA has a limited effect on TNBC but a synergistic effect with drug combinations (Lin et al. 2017; Reinhardt et al. 2018).

In this study, we aimed to examine the potential anti-proliferative effects of combination of NVP-BEZ235 and ATRA in MDA-MB-231 cell line. Cell viability data show that NVP-BEZ235 decreases cell viability in a dose- and time-dependent manner in MDA-MB-231 cells (Fig. 1B). Our results were in consistent with previous studies, were cell viability data show that NVP-BEZ235 reduces cell viability in a dose- and time-dependent manner (Cai et al. 2020), while the viability of ATRA-treated cells is not significantly reduced compared to control cells (Dutta et al. 2010). Furthermore, the combination of NVP-BEZ235 and ATRA showed less toxicity toward healthy epithelial cells when compared to MDA-MB-231 cell line. This might be due to proliferative effect of ATRA toward MCF-10A cell line (Figure S2). The colony formation assay is a technique that allows assessment of long-term proliferative capacity by examining the capacity of a single cell to develop into a large colony through clonal expansion (Rajendran and Jain 2018). To evaluate the effect of our experimental treatments on clonogenicity we have treated MDA-MB-231 cells with NVP-BEZ235, ATRA, and their combinations. Previous studies have shown that treatment with ATRA (Aouad et al. 2017) and NVP-BEZ235 (Cai et al. 2020) causes significant reductions in colony diameter and colony number in the MDA-MB-231 cell line relative to the control group. Consistent with the literature, our findings showed that ATRA and NVP-BEZ235 treated alone showed a significant decrease in the colony-forming ability of MDA-MB-231 cells. In addition, NVP-BEZ235 and ATRA combination treatment exhibited an even stronger inhibitory effect on colony formation of MDA-MB-231 cells.

Invasion assay is used as an important experimental method to determine the ability of cancer cells to penetrate and migrate through the extracellular matrix (ECM), mimicking the initial steps of metastasis (Eccles et al. 2005). Previous studies have shown that ATRA significantly inhibits invasion in MDA-MB-231 cells (Liu et al. 2003; Dutta et al. 2010; Giuli et al. 2020). Studies show that MDA-MB-231 cells treated with 5 μM ATRA alone for 48 h (Mezquita et al. 2018) did not significantl effect on cell viability. Similarly, in our study the number of invasive cells decreased in ATRA alone treated MDA-MB-231 cells was not significant (*p* > 0.05). Furthermore, NVP-BEZ235 alone (*p* < 0.01) and in combination with ATRA (*p* < 0.001) group showed significant decrease in invasive MDA-MB-231 cells. Our results are similar to Cai et al. showing NVP-BEZ235 alone significantly inhibited the invasion of MDA-MB-231 cells (Cai et al. 2020). This observation is consistent with previous studies suggesting that disruptions in the PI3K/Akt pathway and its downstream molecule mTOR play an important role in cancer cell invasion (Samuels et al. 2005; Costa et al. 2018). Additionally, the combination of NVP-BEZ235 with ATRA produced an even more notable inhibitory effect on cell invasion, highlighting the potential synergistic effects of these two compounds in reducing the aggressive nature of TNBC.

The cell cycle is a highly regulated process and a mechanism that plays a critical role in cell growth and proliferation. Dysregulation of the cell cycle underlies the abnormal cell proliferation that characterizes cancer and is a hallmark of many types of cancer (Williams and Stoeber 2012). To evaluate the effect of our experimental treatments on cell cycle progression, we treated cells with the PI3K/mTOR dual inhibitor NVP-BEZ235, ATRA, and their combinations and examined the combined effects of these treatments on the cell cycle profile of MDA-MB-231 cells. Our findings showed that the control group of MDA-MB-231 cells exhibited a dynamic and actively proliferating cell population, consistent with their known aggressive and rapidly proliferating characteristics. Studies have shown that ATRA has minimal effects on apoptosis and cell cycle in MDA-MB-231 cells (Wang et al. 2014; Lin et al. 2017). NVP-BEZ235 is also known to induce cell cycle arrest during the G0/G1 phase in MDA-MB-231 cells (Kuger et al. 2014). Consistent with the literature our results show that ATRA alone had no effect on cell cycle distribution, while NVP-BEZ235 alone and in combination with ATRA showed increase in the G0/G1 phase of cell cycle. An increase in cell density in sub-G0 phase for NVP-BEZ235 alone or in combination with ATRA group was detected. Thus, further studies should be conducted to investigate whether this increase is associated with induction of apoptosis in cells.

In this study, we performed qPCR analysis to evaluate the expression levels of mTOR, BCL-2, Caspase-3 and Caspase-9 genes in MDA-MB-231 cells when treated with 5 μM ATRA, 1 μM NVP-BEZ235 and their combination for 48 h. ATRA treated acute myeloid leukemia cells suppressed mTORC1 activity (Stengel et al. 2022). In our study, we found that the mTOR gene expression level decreased in ATRA treated group and significantly decreased in NVP-BEZ235 alone and combination group when compared to untreated MDA-MB-231 cells. Our results were in consistent with literature (Cai et al. 2020) as NVP-BEZ235 treated cells showed decrease in mTOR gene expression levels. Furthermore, an increase in the expression level of the Caspase-3 gene has been observed in metaplastic Barrett's cells treated with ATRA (Hormi-Carver et al. 2007) and in human neuroblastoma cell line SH-SY5Y treated with NVP-BEZ235 (Çetin et al. 2023). Consistent with the literature, we observed an increase in the expression levels of the Caspase-3 gene following the treatment of MDA-MB-231 cells with ATRA, NVP-BEZ235, and their combinations. Caspase-9 mRNA levels increased in response to retinoic acid treatment in the MCF-7 cell line (Donato and Noy 2005). In the current study, Caspase-9 mRNA level increased in ATRA, NVP-BEZ235 and their combinations treated MDA-MB-231 cells. Studies on the MDA-MB-231 cell line indicate that treatment with NVP-BEZ235 (Li et al. 2018) and ATRA (Sabzichi et al. 2017) did not result in a significant decrease in BCL-2 expression. We observed a decrease (*p* > 0.05) in BCL-2 gene expression of MDA-MB-231 cells when treated with ATRA and NVP-BEZ235. Interestingly, there was a significant decrease in BCL-2 gene expression for combination treated group (Fig. 5B). Changes in expression levels of Caspase-3, Caspase-9 and BCL-2 genes suggest an induction of apoptotic cell death mechanism.

In conclusion, we evaluated the anti-proliferative effects of combination therapy of NVP-BEZ235 and ATRA on the TNBC cell line MDA-MB-231. Our data showed that the combination treatment inhibited cell proliferation, colony formation, and invasion abilities of the cells. The combination therapy causes cell cycle arrest at G0/G1 phase, potentially indicating antiproliferative mechanisms of action. Moreover, qPCR analysis revealed that this combination treatment triggered the apoptotic process by increasing Caspase-3 and Caspase-9 gene expression, whereas it decreased the expression of BCL-2 and mTOR genes. These comprehensive findings indicate that the combination of NVP-BEZ235 and ATRA has a potential anti-proliferative effect on MDA-MB-231 cell lines. These results may provide a strong basis for evaluating this combination therapy in future clinical studies.

## Supplementary Information

Below is the link to the electronic supplementary material.

Supplementary file1 (DOCX 28 KB)

## Data Availability

All source data for this work (or generated in this study) are available upon reasonable request.
