# Supplementary material for: All-trans retinoic acid enhances anti-proliferative effect of dual PI3K and mTOR inhibitor NVP-BEZ235 in triple negative breast cancer
Source: Naunyn Schmiedebergs Arch Pharmacol. 2025 Mar 5;398(8):10855–65. doi: 10.1007/s00210-025-03981-8 (PMC12350528; doi:10.1007/s00210-025-03981-8)
Supplement: Supplementary file 1 — Supplementary file1 (DOCX 28 KB) [file 210_2025_3981_MOESM1_ESM.docx]

**All-trans Retinoic Acid Enhances Anti-Proliferative Effect of Dual PI3K and mTOR inhibitor NVP-BEZ235 on Triple Negative Breast Cancer**

Suranur Ayvaz^1^ and Zeynep Busra Bolat^1,2,3^*

*^1^Molecular Biology and Genetics Department, Hamidiye Institute of Health Sciences, University of Health Sciences-Turkey, Istanbul, 34668, Turkiye*

*^2^Experimental Medicine Research and Application Center, Validebag Research Park, University of Health Sciences, Uskudar, Istanbul, 34662, Turkiye*

*^3^Department of Molecular Biology and Genetics, Faculty of Engineering and Natural Sciences, Istanbul Sabahattin Zaim University, Istanbul, 34303 Turkiye*

***Corresponding author:**

Zeynep Busra Bolat, PhD, Molecular Biology and Genetics Department, Hamidiye Institute of Health Sciences, University of Health Sciences- Turkey, Istanbul, 34668, *Turkiye.*

E mail address: zeynepbusra.bolat@sbu.edu.tr

**Figure S1.** Effects of combinatorial treatment of NVP-BEZ235 (N) and ATRA (A) and DMSO (0.1%) on the MDA-MB-231 cell line for 48 h. The cell viability was determined by MTS assay. Data are presented as mean ± standard deviation (n = 3) ( **p < 0.01, ***p < 0.001).

**Figure S2.** Effects of ATRA, NVP-BEZ235 and their combination on the MCF-10A cell line. Percentage of viable MCF-10A cells after treatment with 5 µM ATRA, 1 µM NVP-BEZ235 and their combination for 48 h. The cell viability was determined by MTS assay. Data are presented as mean ± standard deviation (n = 3) (*p < 0.05, **p < 0.01, ***p < 0.001, ****p < 0.0001).

**Table S1.** Primers sequences used in qRT-PCR.

| Gene | Primer Sequence (5' - 3') |
| --- | --- |
| mTOR | F: CTGGGACTCAAATGTGTGCAGT |
|  | R: GAACAATAGGGTGAATGATCCGGG |
| BCL-2 | F: AGAGCAACCCAATGCCCGC |
|  | R: CAACGAGGGGCCTGAGAGG |
| Caspase-3 | F: GGGAGCAAGTCAGTGGACTC |
|  | R: CCGTACCAGAGCGAGATGAC |
| Caspase-9 | F: CGGTGACGCAAGAGCGAATC |
|  | R: GATCAGCTGCCTGGCCTGAT |
